# Resveratrol and Its Nanoformulation Attenuate Growth and the Angiogenesis of Xenograft and Orthotopic Colon Cancer Models

**DOI:** 10.3390/molecules25061412

**Published:** 2020-03-20

**Authors:** Thangirala Sudha, Ali H. El-Far, Deena S. Mousa, Shaker A. Mousa

**Affiliations:** 1 Pharmaceutical Research Institute, Albany College of Pharmacy and Health Sciences, Rensselaer, NY 12144, USA; Sudha.Thangirala@acphs.edu; 2 Department of Biochemistry, Faculty of Veterinary Medicine, Damanhour University, Damanhour 22511, Egypt; ali.elfar@damanhour.edu.eg; 3 Jonathan Edwards College, Yale University, New Haven, CT 208220, USA; deena.mousa@yale.edu

**Keywords:** colon cancer, resveratrol, nanoformulation, xenograft, orthotopic model

## Abstract

Cancer is a multifactorial disorder that induces mortality worldwide, and the colorectal type is the third most common cancer globally. Resveratrol (RSV) is a natural compound with an effective anticancer effect, especially against colorectal cancer, and therefore numerous studies recommended its use in colorectal cancer prevention and treatment. The current study investigated the effect of either RSV or its nanoformulation (NP-RSV) on the growth and vascularity of xenograft and orthotopic mice models in colon cancer (COLO205-luc). Both RSV and NP-RSV induced significant reductions in tumor growth and the hemoglobin percentages of the tumor mass, but NP-RSV showed greater bioavailability and efficacy than RSV. Generally, we recommend using NP-RSV as a therapeutic to control colon cancer.

## 1. Introduction

About 14 million cases of cancer and 8.2 million deaths from cancer have been recorded per year; therefore, cancer is one of the world’s major health problems [1]. Today, the fourth most deadly cancer in the world is colorectal cancer, and obesity and smoking are increasing the risk of colorectal cancer [2]. Colon cancer is a common malignancy in the United States [3]. The main risk factors for colorectal cancer are family history and lifestyle factors such as consumption of red meat, tobacco smoking, and alcohol intake along with environmental factors [4].

Several trials have been established to study the control of colorectal cancer using natural compounds, such as resveratrol (RSV), which is considered to be a promising natural therapy for colorectal cancer prevention and treatment [4]. RSV (trans-3,4′,5-trihydroxystilbene) is in pigmented vegetables and fresh fruits (mainly in grapes) as well as in dried nuts such as peanuts [5]. RSV is a stilbenoid polyphenol that has two phenol rings linked to each other by an ethylene bridge. It has numerous biological activities, including antioxidant, antitoxic, antimicrobial, and anticancer potentials [6]. RSV has been reported to have chemopreventive and chemotherapeutic activities [7] through the induction of cell cycle arrest [8], and the downregulation of the mammalian target of rapamycin (mTOR) [9], nuclear factor-κB (NF-κB) [8,10,11], B-cell lymphoma 2 (Bcl2) [10], and cyclooxygenase-2 (COX-2) [12]. Moreover, RSV significantly decreased the proliferation of human colon cancer cells (LoVo) by the activation of p38-MAPK [13]. RSV inhibited the proliferation and angiogenesis and induced apoptosis in colon cancer cells (HCT116 and Caco2) [14].

Numerous studies have showed an enhancement in the bioavailability of RSV by nanoformulations and an increase in the sensitivity of cancer cells to chemotherapy. Zhao et al. [15] found an increase in the sensitivity of breast cancer-bearing mice to co-delivery of doxorubicin (Dox) and RSV via poly glyco-lactic acid (PLGA) nanoparticles (NPs) to overcome Dox resistance. Moreover, RSV oral bioavailability was increased 10 times when loaded in casein NPs [16]. RSV-loaded PLGA NPs induced excessive apoptosis of the prostate cancer cell line (LNCaP) [17]. Another type of RSV NP was studied by Wang et al. [18] who formulated RSV-loaded solid lipid NPs (SLNs) and found an increase in the ratio of Bax/Bcl-2 and decreases in the expression of cyclin D1 and c-Myc in MDA-MB-231 cells.

RSV and its nanoformulations (NP-RSV) have promising anticancer effects. The current study was conducted to investigate the antiproliferative and anti-angiogenic effect of RSV and its nanoformulation prepared by encapsulating RSV into PLGA-polyethylene glycol (PEG) NPs coated with chitosan and tested against human COLO205-luc colon cancer in xenograft and orthotopic implantation models of athymic mice.

Most previous studies with different RSV nanoforumlations from what we used were mainly carried out in vitro with cancer cells, while in our current investigation we tested the anticancer and anti-angiogenesis efficacy of nano-RSV coated with muco-adhesive chitosan for improved bioavailability.

## 2. Results

The loading efficiency of RSV in the nanoformulation was 85%, with 12% loading *w*/*w* (RSV/NP-RSV).

**Figure 1:**
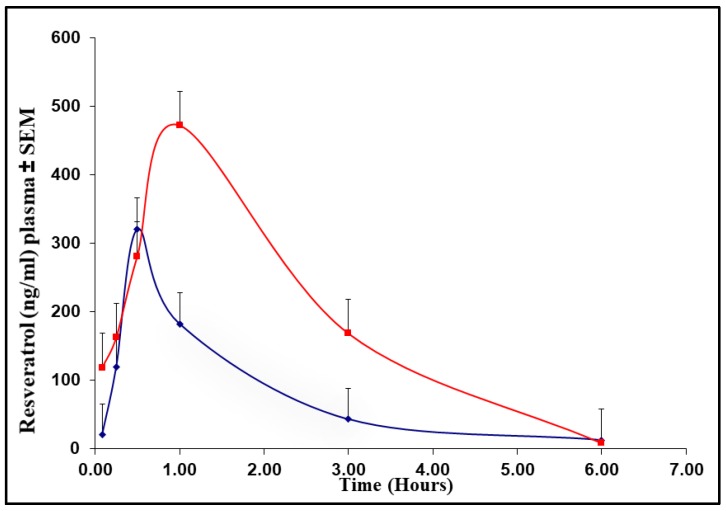
Plasma resveratrol (RSV) concentration (ng/mL)–time curve in mice (*n* = 3) treated with RSV (blue line) or resveratrol nanoformulation (NP-RSV) (red line) at 4 mg/kg.

Greater bioavailability was demonstrated for NP-RSV versus RSV administered to mice at 4 mg/kg, RSV equivalence, based on the area under the curve of plasma levels of RSV over time (Figure 1). Mice from the RSV and NP-RSV groups showed no toxicity signs.

**Figure 2:**
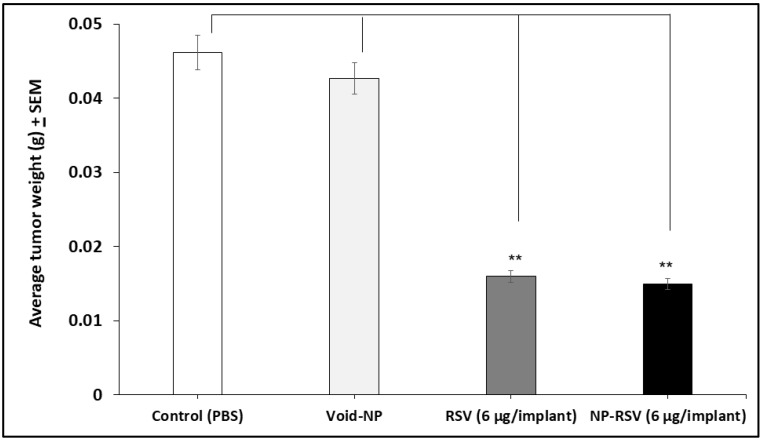
Resveratrol (RSV) and its nanoformulation (NP-RSV) reduced subcutaneous COLO205-luc tumor weight. Four implants/mouse subcutaneously, 3 animals per group. ** *P* < 0.01.

**Figure 3:**
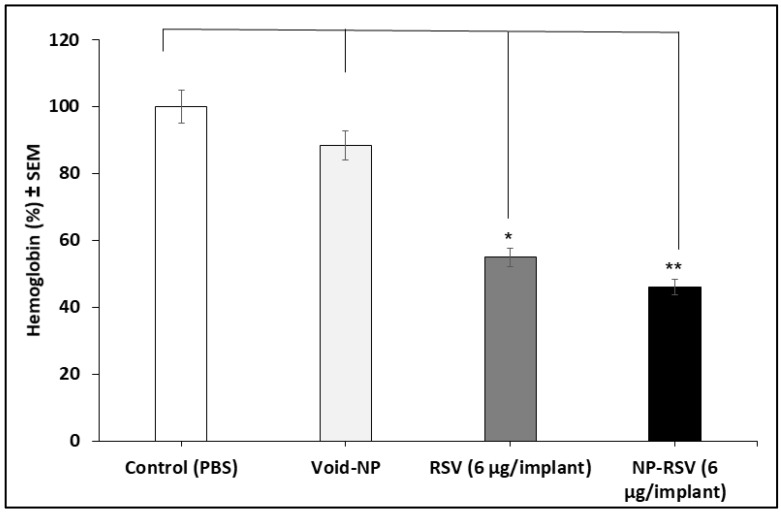
Resveratrol (RSV) and its nanoformulation (NP-RSV) reduced subcutaneous COLO205-luc tumor hemoglobin percentages. Four implants/mouse subcutaneously, in each of the 3 animals per group. * *P* < 0.05; ** *P* < 0.01.

Both RSV and NP-RSV induced a significant (*P* < 0.01) reduction in tumor weight (Figure 2). Also, the hemoglobin (Hb) percentages in COLO205-luc tumors were significantly reduced when treated with RSV (*P* < 0.05) and NP-RSV (*P* < 0.01) (Figure 3).

**Figure 4:**
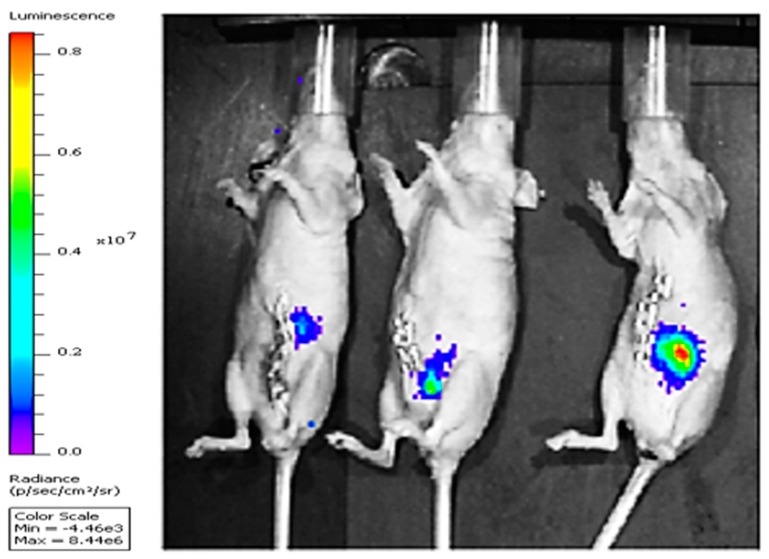
Representative IVIS images of mice with orthotopic implants of COLO205-luc after 3 days of implantation in cecum.

**Figure 5:**
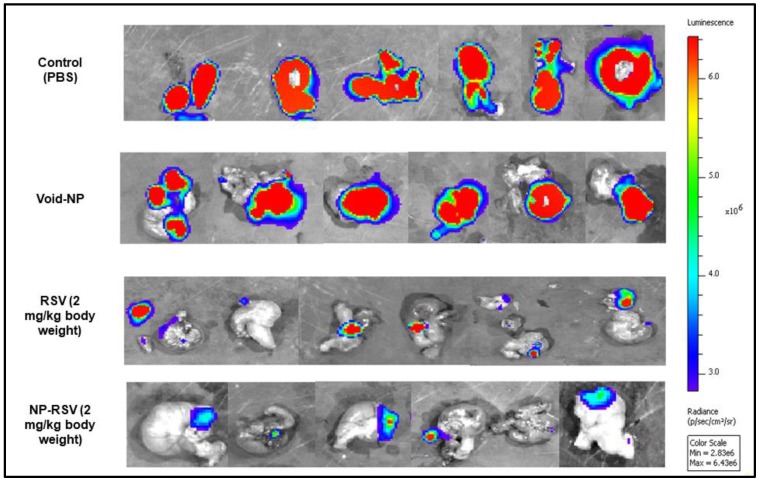
IVIS images (ex vivo) of the orthotopic COLO205-luc tumors after 3 weeks of treatment with resveratrol (RSV) and its nanoformulation (NP-RSV). High signal intensity, red color areas indicate increased viability of cancer cells, while blue color areas indicate the lowest viability or necrosis. A light blue to green color indicates low viability.

**Figure 6:**
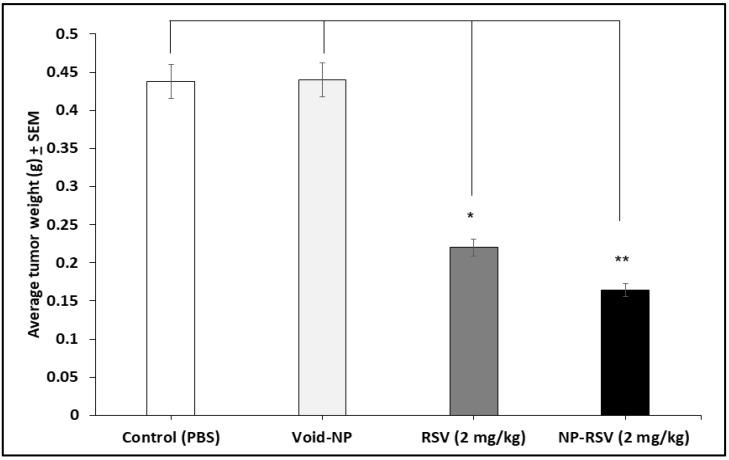
Resveratrol (RSV) and its nanoformulation (NP-RSV) reduced the weight of orthotopic colon tumor (COLO205-luc). * *P* < 0.05; ** *P* < 0.01.

**Figure 7:**
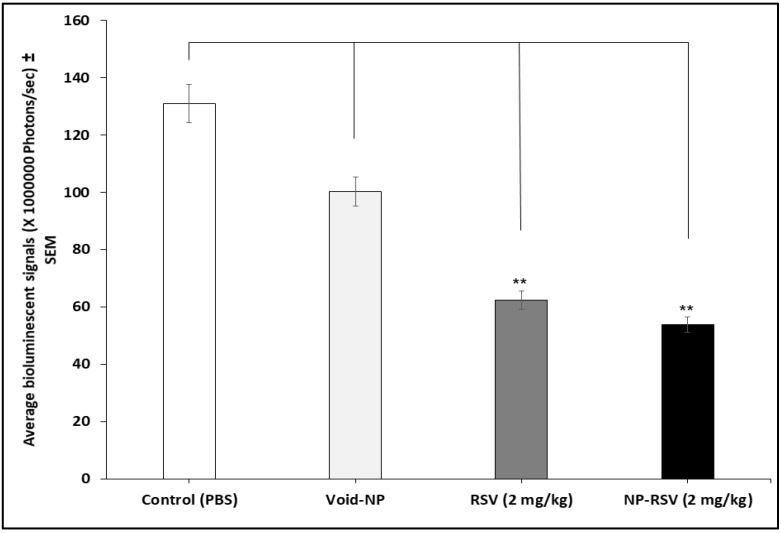
Resveratrol (RSV) and its nanoformulation (NP-RSV) reduced bioluminescent signals of orthotopic colon tumor (COLO205-luc). ** *P* < 0.01.

Figure 4 shows the bioluminescence signals of orthotopic implants of COLO205-luc tumors in cecum using IVIS. Moreover, Figure 5 and Figure 6 show the significant reduction in orthotopic implanted COLO205-luc tumor growth in cecum in response to RSV (*P* < 0.05) and NP-RSV (*P* < 0.01) treatment for 24 h, and the bioluminescent signals of orthotopic colon tumor (COLO205-luc) were reduced by the same extent (*P* < 0.01) (Figure 5, Figure 6 and Figure 7). Treatments were well tolerated by the mice, and no difference in animal weight was found between the experimental groups.

## 3. Discussion

Resveratrol is considered to be a promising anticancer drug for chemoprevention or therapy for colon cancer, targeting numerous cellular molecules, such as AKT serine/threonine kinase 1 (AKT1), AKT2 [19], and AKT/GSK-3β/Snail signaling pathway [20], and increasing ROS production [21]. In the current study, RSV at a dose of 6 µg/implant significantly reduced the tumor weight of s.c. (subcutaneously)-implanted COLO205-luc in athymic mice. Lee et al. [7] stated that RSV attenuated the growth of human ovarian cancer (PA-1) cell xenograft through the downregulation of eukaryotic elongation factor 1A2 (eEF1A2), leading to a marked decrease in PA-1 proliferation. Furthermore, RSV upregulated the expression of caspase-9 and caspase-3 in an implanted human primary ovarian cancer cell (SKOV3) in nude mice [22]. RSV effectively suppressed the growth of pancreatic cancer (PaCa) in an orthotopic mouse model through the downregulation of NF-κB, cyclin D1, COX-2, intercellular adhesion molecule-1 (ICAM-1), matrix metallopeptidase-9 (MMP-9), and survivin [8]. In addition, RSV upregulated the mRNA expression of p53 and extracellular signal-regulated kinase (ERK) in squamous cell carcinoma (A431) xenografts in nude mice [23].

The Hb percentage of tumor mass is an indicator of the angiogenesis process [24]. Here, RSV significantly reduced the Hb percentages of tumor mass, indicating the anti-angiogenetic effect of RSV against implanted COLO205-luc. Similar results were reported by Hu et al. [25], who found a significant anti-angiogenetic effect of RSV alone or in combination with ginkgetin, a biflavone from Ginkgo biloba leaf, through the downregulation of vascular endothelial growth factor (VEGF)-mediated angiogenesis. Moreover, RSV induced apoptosis of in vivo implanted breast cancer cells (MDA-MB-231) through a significant reduction in extracellular levels of VEGF [26]. Moreover, RSV significantly inhibited tumor growth and angiogenesis in nude mice through a reduction in micro-vessel density [27].

Orthotopic transplantation has been done by transplantation of a tumor into the same organ or other recipient animals to study the tumorigenic properties and metastatic ability of human cancer [28]. In our study, RSV reduced the growth and bioluminescent signals of orthotopic colon tumor (COLO205-luc). In another study in xenograft mice, a combination of RSV and oxaliplatin was more effective at inhibiting tumor growth than RSV and oxaliplatin alone of implanted colon cancer [29,30].

The nanoformulation of polyphenol compounds in hydrophobic polymer is expected to enhance their solubility, stability, and increase oral bioavailability along with the extension of their half-life [31,32,33]. This idea was confirmed by an in vivo study, where RSV nanoencapsulation significantly increased bioavailability and the therapeutic efficacy of RSV against colon cancer cells (HT29) [34]. The results of the current study reveal that NP-RSV is more potent than RSV in transplanted COLO205-luc through a significant reduction in tumor growth, Hb percentages, and cecal orthotopic transplant. This could be interpreted as enhancing the bioavailability of RSV by nanoformulation. Similarly, Zhao et al. [15] stated that Dox and RSV co-encapsulated in modified PLGA NPs significantly inhibited Dox-resistant tumor growth in MDA-MB-231 and MCF-7 tumor-bearing mice without causing significant systemic toxicity. Moreover, RSV nanocomposite had a high anticancer efficacy against mouse colon cancer cells CT26 in vitro and in a xenograft mouse model with less tissue toxicity [35]. Previous in vitro studies showed that PLGA-PEG-COOH NPs of RSV significantly improved the efficacy of RSV toward both the androgen-independent DU-145 and hormone-sensitive LNCaP prostate cell lines compared with free RSV [36].

## 4. Materials and Methods

### 4.1. Cell Line and Reagents

The human colon cancer cell line (COLO205-luc) expressing firefly luciferase was provided by MD Anderson Cancer Center (Houston, TX, USA). Cell culture reagents and standard hemoglobin, Drabkin’s reagent, resveratrol (RSV), poly glycol-lactic acid (PEG), and other common reagents were purchased from Sigma (St. Louis, MO, USA). D-Luciferin potassium salt was purchased from Caliper Life Sciences (Hopkinton, MA, USA). Matrigel was purchased from BD Bioscience (San Jose, CA, USA).

### 4.2. Synthesis of PLGA-PEG Nanoparticles Coated with Chitosan and RSV Encapsulation

**Figure 8:**
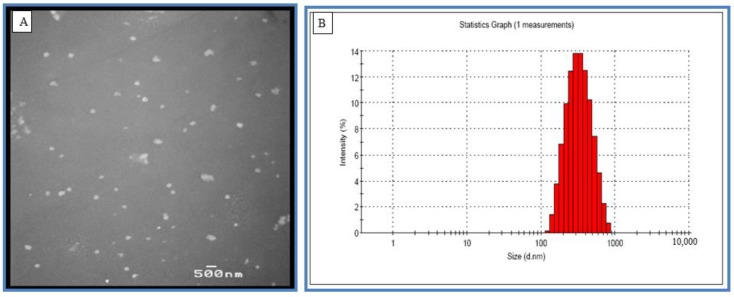
Particle size determination of resveratrol nanoformulation (NP-RSV) Using (**A**) transmission electron microscopy (TEM) and (**B**) zeta size analyzer. The average nanoparticle encapsulating RSV ranged from 200–220 nm.

In our previous studies, we synthesized PEG poly glycol-lactic acid-based nanoparticles (NPs) encapsulating RSV (Figure 8) and evaluated their capability as an efficient formulation. Thus, in this study, we synthesized RSV encapsulated in poly glyco-lactic acid-polyethylene glycol (PLGA-PEG) NPs (with an average size of 8000 Daltons) and coated them with chitosan.

Briefly, 200 uL of PLGA-PEG (80 mg/mL DMSO) and 100 µL of RSV at 100 mg/mL DMSO were mixed together and then added dropwise under constant magnetic stirring to 20 mL of 2% *w*/*v* PVA solution. The solution was then sonicated for 30 s using a probe sonicator. Immediately following sonication, the solution was stirred using magnetic stirring for at least 3 h, and it was then dialyzed for purification. Then, 2% chitosan acetate (with an average size 4000 daltons) was used for coating the negatively charged carboxylic acid surface until the zeta potential approached + 5–10 mv.

Finally, this solution containing the PLGA-PEG NPs encapsulating RSV was lyophilized in the presence of 1–2% sucrose to get the nanoformulation in a powder form. These NPs were characterized with particle size analysis and transmission electron microscopy. The total amount of the RSV encapsulated in the NPs was determined with HPLC-MS/MS. The average size of NP-RSV was estimated using a zeta size analyzer and transmission electron microscopy (TEM) ranging from 200–220 nm (Figure 8).

### 4.3. Determination of Loading Level of Resveratrol in Nanoformulation

To determine the RSV loading level, a six-point standard curve was constructed from RSV standard solutions at working concentrations of 0.3125, 3.125, 6.25, 12.5, 25, and 50 µg/mL. IN total, 20 µL NP-RSV and 80 µL methanol were vortexed for 3 min and then sonicated for 1 min. The resulting solution was diluted 20-fold with 70% methanol containing 0.1% formic acid, and 20 µL was injected onto HPLC-MS/MS (Applied Biosystems, MDS SCIEX Ontario, Canada). RSV separation was carried out on a C18 column (Waters, Milford, MA, USA), 150 × 3.0 mm, 5 µm. The mobile phase consisted of 70% methanol and 0.1% formic acid at 0.5 mL/min. The detection wavelength was set at 306 nm.

### 4.4. Cells and Cell Culture

COLO205-luc cells were grown in Dulbecco’s Modified Eagle’s medium (DMEM) supplemented with 10% fetal bovine serum, 1% penicillin, and 1% streptomycin. The cells were cultured at 37 °C in a humidified atmosphere of 5% CO_2_ until confluence of 80–90 % and were treated with 0.25% (*w*/*v*) trypsin/EDTA to release cells from the culture flask. After washing the cells with culture medium, they were suspended in DMEM (free of phenol red and fetal bovine serum) and counted.

### 4.5. Animals

Athymic female mice (4–5 weeks of age, 15–20 g weight) were purchased from Taconic Biosciences (Germantown, NY, USA). All animal studies were conducted at the animal facility of the Veteran Affairs Medical Center, Albany, NY, USA in accordance with and approved (Protocol number 545017) by current institutional guidelines for humane animal treatment. The mice were maintained under specific pathogen-free conditions and housed under controlled of temperature (20–40 °C) and humidity (60–70%) and 12 h light/dark cycle with ad libitum access to water and food. The mice were allowed to acclimatize for 5 days before the study.

### 4.6. Tumor Xenograft Model for Angiogenesis

COLO205-luc cells were injected subcutaneously (s.c.) into the right front axilla of the mice (5 × 10^6^ cells/0.1 mL/mouse). The cells were suspended in PBS (control), void NP, RSV (6 µg/implant), or NP-RSV (6 µg/implant) and were injected s.c. There were 4 implants per mouse and each group had 3 animals. On day 7, mice in all groups were sacrificed and the tumor masses were collected and weighed and Hb concentrations analyzed.

### 4.7. Assessment of Resveratrol in Plasma with LC-MS/MS

Plasma samples were assayed for RSV with a liquid chromatography tandem mass spectrometry (LC-MS/MS) approach using an API 4000 triple-quadrupole mass spectrometer (Applied Biosystem MSD Sciex, Carlsbad, CA, USA). Briefly, 30 µL of mouse plasma was added into an equal amount of normal saline and 300 µL tertiary-butyl methyl ether (tBEM) and vortexed thoroughly for 10 min. After centrifugation at 20,000× *g* for 10 min, the upper layer solvent was dried under a nitrogen stream. The residue was resuspended with 70% methanol and 0.1% formic acid and 20 µL was injected onto the LC-MS/MS. The sample was chromatographed on a Sunfire C18 column (Waters), 50 × 3.0 mm. The mobile phase was 70% methanol and 0.1% formic acid at 0.4 mL/min. The tandem mass-spectrometer was operated in negative electrospray mode with the ion spray voltage set at 4.2 KV and temperature 600 °C. RSV was detected using multiple-reaction monitoring, and quantitation was achieved by monitoring mass transition 227.0/184.9 (*m*/*z*). All data were acquired and processed with Analyst 1.4.2 software (Applied Biosystem MSD Sciex). The limit of detection was 35 pg and the limit of quantitation was 3.9 ng/mL plasma.

### 4.8. Orthotopic Xenograft Model

We followed the method described by Chan et al. [37] with slight modification for developing an orthotopic model for colon cancer. In brief, small pouches were created at the tip of the cecum and subcutaneous equal size tumor fragments, as described below, were enveloped within the cecal pouches and tied with 3–0 silk thread.

We used s.c. COLO205-luc tumors for orthotopic implantation. Cancer cells were injected s.c. into the right front axilla of the mice with 0.2 × 10^6^ total cells in 100 uL of 50% Matrigel per implant. Seven athymic female mice were injected with COLO205-luc cells s.c. and had 4 implants each. Animals were terminated after 3 days and the tumors were collected in medium for orthotopic implantation.

Collected tumors were dissected into pieces of equal size and were implanted into the cecal pouch of athymic female mice under anesthesia. On day 3, in vivo imaging of the bioluminescence signal of COLO205-luc tumors was detected using the IVIS Spectrum In Vivo system (Perkin Elmer, Waltham, MA, USA). Then, s.c. treatment started daily with PBS, Void-NP, RSV (2 mg/kg body weight) or NP-RSV (2 mg/kg body weight) and 6 animals per group. After 3 weeks, the animals were terminated, tumors were collected and weighed, and the tumor bioluminescent signal intensity (ex vivo IVIS imaging) was measured. Bioluminescence was quantified as photons/second for each region of interest.

### 4.9. Statistical Analysis

Statistical analysis was performed using a one-way ANOVA for each experimental group with its respective control group. Statistical differences approaching *P* < 0.05 were considered statistically significant.

## 5. Conclusions

The results of the current study reveal that NP-RSV significantly reduced the tumor growth of implanted COLO205-luc in both subcutaneous and cecal orthotopic implantation mouse models. Furthermore, NP-RSV significantly reduced the angiogenesis of COLO205-luc that was monitored with the reduced percentage of tumor Hb. These results reveal the promising anticancer and antiangiogenic effect of NP-RSV.
